# A Novel Approach to the Analysis of Under Sleeper Pads (USP) Applied in the Ballasted Track Structures

**DOI:** 10.3390/ma13112438

**Published:** 2020-05-26

**Authors:** Artur Zbiciak, Cezary Kraśkiewicz, Anna Al Sabouni-Zawadzka, Jan Pełczyński, Sławomir Dudziak

**Affiliations:** 1 Institute of Roads and Bridges, Faculty of Civil Engineering, Warsaw University of Technology, Al. Armii Ludowej 16, 00-637 Warsaw, Poland; a.zbiciak@il.pw.edu.pl; 2 Institute of Building Engineering, Faculty of Civil Engineering, Warsaw University of Technology, Al. Armii Ludowej 16, 00-637 Warsaw, Poland; a.sabouni@il.pw.edu.pl (A.A.S.-Z.); j.pelczynski@il.pw.edu.pl (J.P.); s.dudziak@il.pw.edu.pl (S.D.)

**Keywords:** under sleeper pads, vibration damping, rheological model, finite element method

## Abstract

The present paper is dedicated to the analysis of under sleeper pads (USP), which are resilient elements used in ballasted track systems as vibration isolators. Four types of USP are considered. The authors present the results of laboratory tests, which are then used as input values for the finite element (FE) and mechanical model of the structure. A special focus is put on the description of an original four-degree-of-freedom (4DoF) mechanical model of the system that includes a fractional rheological model of USP. Using the proposed approaches, the dynamic characteristics of under sleeper pads are determined, and conclusions on vibration isolation effectiveness are drawn.

## 1. Introduction

Under sleeper pads (USP) are resilient elements applied, among other things, in the ballasted track systems to protect the structure as well as its surroundings against vibration caused by the rolling stock [1,2]. They protect the ballast by reducing the stress, as they increase the contact area between the sleeper and the ballast layer. Moreover, USP improve the stability of the track and increase the technical lifespan of the structure.

The pads are made from elastomeric materials, among which two variants can be specified: polyurethane-based pads (with closed or open pores) and rubber-based pads (blends of natural rubber and/or synthetic rubber). They are installed under the sleepers or turnout bearers and can cover either the full surface or only part of it (the effective area of the load transmission). USP installation can be realized in two ways: they can be put into formworks before concreting the sleepers (or turnout bearers) or glued to the ready elements using a fast hardening adhesive.

Thanks to a wide variety of available USP, which can differ both geometrically and mechanically, they can be applied in various track systems, including high-speed railways and railways with high axle loads. In the design of the system with USP, two main parameters should be taken into account: the maximum deflection of the rail and the natural frequency of the structure.

There are many works focused on the behavior of USP and other elastic vibration isolators applied in the ballasted track structures. State-of-the-art resilient elements used in railway systems are presented in [3], where a variety of features with regard to track stiffness, noise, vibrations, geometry degradation, etc. is discussed. In [4], a laboratory research on the mechanical properties of selected resilient elements is presented. The authors analyze different configurations of the track section and indicate solutions that decrease the track stiffness and enhance the capacity to dissipate energy. Another paper [5] is dedicated to the rail pads manufactured from deconstructed end-of-life tires. The influence of the pads’ thickness on their mechanical behavior under loads simulating a moving train is assessed. The same product—end-of-life tires—may also be used to manufacture a crumb rubber that can be applied as elastic aggregates mixed with ballast particles [6]. The presented solution should protect the ballast layer against fast degradation and enhance its capacity for energy dissipation. Another possibility for reducing the track settlements lies in the application of polymer reinforced ballast, as proposed in [7]. A study on the vibration attenuation at rail joints using resilient USP attached to concrete sleepers is presented in [8]. Another work [9] discusses the possibility of controlling the track subsidence with the use of USP. The authors in [10] prove that the application of USP can lead to the reduction of the maintenance requirements and whole-life costs for the track. In [11], the role of USP in decreasing the ballast degradation and reducing the permanent deformation of the track is discussed. Papers [12,13,14,15,16,17,18] are dedicated to the mechanical modeling of railway track structures with elastic vibration isolators, such as the under sleeper pads considered in the present work.

In the present paper, a novel approach to the analysis of ballasted track systems retrofitted with resilient under sleeper pads is proposed. In the first part, the authors discuss the results of laboratory tests performed to determine the static and dynamic characteristics (bedding moduli) of the analyzed USP samples. The values obtained from these tests were afterwards used as an input for the finite element (FE) and mechanical model of the structure. The FE model of the considered ballasted track system with USP is presented in the following section. It is a 2D model in plane strain, which provides the static and dynamic characteristics of the analyzed structures with resilient pads. For each of the four considered pad materials, three characteristics were determined: the magnitude of vertical displacement of the left rail head in the frequency domain, transmissibility function and insertion loss function.

The last section of this paper is dedicated to the novel mechanical model of ballasted track structures with resilient elements. Typically, in the analyses of railway track structures with vibration isolators, discrete mechanical models with few degrees of freedom are used. In the present paper, the authors present a mechanical model that has four degrees of freedom (4DoF) corresponding to four material points: rail and rolling stock, sleeper, ballast and soil subgrade. The elastic and dissipative elements are modeled with rheological Kelvin–Voigt systems, which characterize the properties of the rail pad, ballast and subsoil. Moreover, the isolated system contains an additional level representing the under sleeper pad, whose rheological viscoelastic model is composed of two springs and a fractional element (spring-pot). The proposed mechanical model precisely describes the behavior of the structure and is more accurate than the standard system.

It is shown that by combining two approaches in the analysis of track structures, a full information on the structural behavior and the effectiveness of vibration isolation can be obtained. While the FE model is more detailed and does not require adopting effective masses of particular structural elements, the 4DoF model is more synthetic and makes it possible to formulate simple analytic expressions that describe the properties of the analyzed systems with the use of Bode plots.

## 2. Laboratory Tests of USP

There are two basic parameters that characterize the elastic properties of USP: the static and dynamic bedding moduli. They affect the effectiveness of damping the vibration transmitted to the surroundings of the railway tracks. The higher the values of the bedding moduli are, the less effective the protection against vibration is. However, it should be noted that the application of very soft pads leads to bigger vertical rail deflections which, on the other hand, cause fatigue phenomena in the rail and in other elements of the track structure.

The static bedding modulus $C_{\mathrm{stat}}$ can be defined as the ratio of the static load (applied to the sample with a certain cross-sectional area) to the sample deflection caused by this load. It characterizes the deflection of the track system under a non-moving rolling stock. The value of the static bedding modulus of USP depends on the applied load, but the relationship is not linear. Therefore, it should be determined for various load ranges depending on the pad application: tram, subway, city railway or inter-city railway. Such ranges are defined in the standard EN 16730 [19] for four track categories: TC1, TC2, TC3 and TC4.

The dynamic bedding modulus $C_{\mathrm{dyn}}$ is the ratio of the dynamic load with a specified value and frequency (applied to the sample with a certain cross-sectional area) to the sample deflection caused by this load. It characterizes the deflection of the track system under a moving rolling stock. The value of the dynamic bedding modulus depends not only on the applied load, as in the case of the static modulus, but also on its frequency, and it should therefore be tested in the conditions of a normalized load and frequencies specified in the standard EN 16730 [19].

In this paper, the results of the tests performed on four USP samples are presented:

- 018—a polyurethane-based pad with a styrene-butadiene rubber (SBR)—thickness 13 mm;
- 036—a styrene-butadiene rubber-based pad (SBR)—thickness 5 mm;
- 075—a styrene-butadiene rubber-based pad (SBR)—thickness 10 mm;
- 095—a polyurethane-based pad—thickness 8 mm.

**Figure 1:**
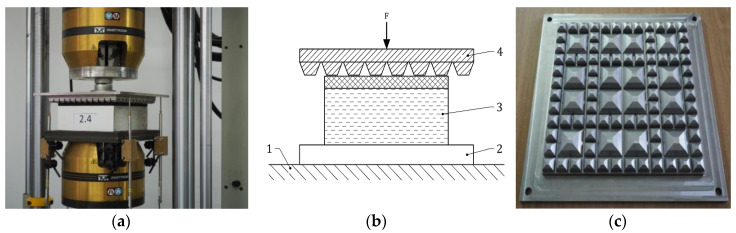
Testing procedure: (**a**) under sleeper pad (USP) sample attached to the concrete block; (**b**) test scheme (1—non-deformable support, 2—steel plate, 3—USP on concrete block, 4—GBP); (**c**) geometric ballast plate (GBP).

The tests were carried out using USP samples with the dimensions 250 mm × 250 mm × USP thickness that were attached to concrete blocks 250 mm × 250 mm × 100 mm (Figure 1a). The samples were loaded via a geometric ballast plate (GBP) (Figure 1c) and supported by non-deformable smooth steel plates with a sanding disc (K240 grit on a rigid linen backing cloth). The static and dynamic load was applied by the INSTRON 8802 hydraulic fatigue testing system (Instron, Norwood, MA, USA). The deflections were measured with four displacement inductive sensors (WA-T type by HBM, Hottinger Baldwin Messtechnik GmbH, Darmstadt, Germany) together with the HBM Spider8 data acquisition and signal conditioning system and dedicated software - Catman AP (version 3.4). The test scheme is presented in Figure 1c.

The values of the static and dynamic bedding moduli obtained from the laboratory tests of the four considered USP are given in Table 1.

Based on the values of the bedding moduli, the pad USP 018 can be classified as very soft, USP 075 as soft, USP 095 as medium and USP 036 as stiff. The softest pad, USP 018, would be most effective for vibration isolation, but at the same time it would cause significant rail deflections. The stiffest pad, USP 036, on the other hand, is not an effective vibration isolator.

The values obtained from the laboratory tests are used further in the analyses of the ballasted track structures with resilient under sleeper pads.

## 3. Finite Element Model of the Structure

**Figure 2:**
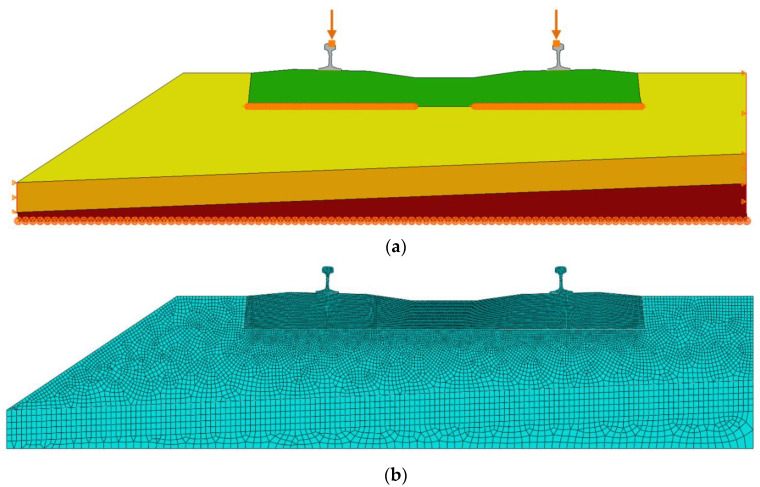
Finite element (FE) model of the ballasted track structure: (**a**) Geometry, boundary conditions, loads, concentrated masses and spring-dashpot systems (layers: grey—rails, green—sleeper, yellow—ballast, orange—protection layer, brown—soil subgrade); (**b**) FE mesh.

The FE analysis of the structure was performed in the Abaqus software (Abaqus/CAE 2016). A numerical model of the analyzed ballasted track system is shown in Figure 2. It is a 2D model in plane strain, in which 3- and 4-node finite elements with linear shape functions and a reduced integration were applied. To satisfy the FE method convergence condition, three mesh densities were analyzed. There are 20,769 nodes and 19,656 elements in the model. The boundary conditions were applied at two vertical edges of the structure, disabling the horizontal movement—displacements in the horizontal direction were restrained along both edges (Figure 2a).

The values of the material parameters used in the FE analysis are given in Table 2. The values were adopted based on the literature study [17], DIN standards [20,21] and the authors’ own experience.

The values of the loss factor for the rail, sleeper and ballast ($\eta_{o}$) were taken from the DIN standard [20]. For the protection layer and the soil subgrade, the parameter $\eta_{u}$ was calculated as:

(1) $$\eta_{u}=\frac{d_{u}}{\sqrt{k_{u}\cdot m_{\mathrm{eff}}}},$$

where: $k_{u}=15\times 10^{8}\;N/m$, $d_{u}=1.2\times 10^{6}\;\mathrm{Ns}/m$, $m_{\mathrm{eff}}=2600\;\mathrm{kg}$ (values from [20]).

Moreover, a part of the soil subgrade was modeled as a parallel combination of springs-dashpot systems (Figure 2a), with the values of the stiffness and damping coefficients corresponding to $k_{u}$ and $d_{u}$. In a similar way, two rail pads were modeled, with the parameters $k_{p}=1\times 10^{8}\;N/m$ and $d_{p}=1\times 10^{5}\;\mathrm{Ns}/m$. Additionally, two concentrated masses—m=3400 kg each—were applied at the top of the rail heads to simulate the rolling stock (Figure 2a).

**Figure 3:**
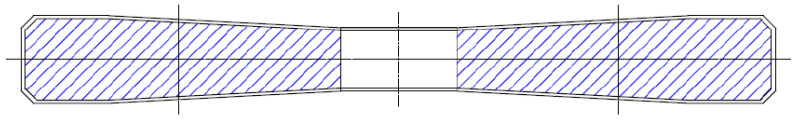
Effective area of USP in the sleeper PS-94.

The analyzed model included under sleeper pads that were modeled similarly to the rail pads, using the spring-dashpot systems in parallel (Figure 2a). The USP parameters were calculated using the values of the static $C_{\mathrm{stat}}$ and dynamic $C_{\mathrm{dyn}}$ bedding moduli obtained from the laboratory tests, and the following formulae:

(2) $$k_{\mathrm{USP}}=C_{\mathrm{stat}}\cdot A_{\mathrm{eff}},$$

(3) $$d_{\mathrm{USP}}\left(f\right)=\frac{\sqrt{{\left(C_{\mathrm{dyn}}\left(f\right)\right)}^{2}-{\left(C_{\mathrm{stat}}\right)}^{2}}}{2\pi f}\cdot A_{\mathrm{eff}},$$

where: f∈{8,10,12.5,16,20,24} Hz, and the effective area of the sleeper is $A_{\mathrm{eff}}=2\times 2703\;{\mathrm{cm}}^{2}=5406\;{\mathrm{cm}}^{2}$ (Figure 3).

Two types of calculations were performed within the FE method: a static analysis under a unit load and a steady-state dynamic analysis under the harmonic excitation with a unit amplitude. In both cases, two concentrated forces of 1 N were applied to the rail heads (one force to each rail). The dynamic analysis was performed using a modal method with the considered range of frequencies 0 to 1000 Hz.

**Figure 4:**
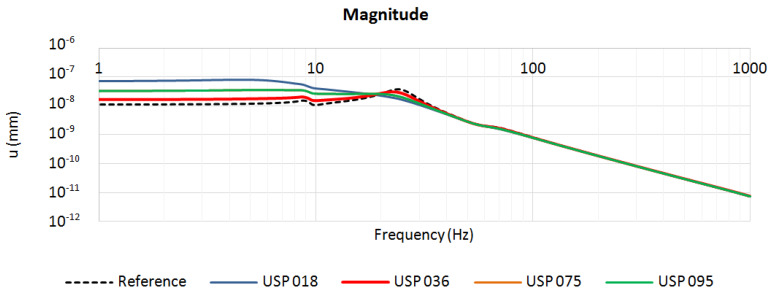
Magnitude of the rail head vertical displacement: reference system and systems with four analyzed USP.

**Figure 5:**
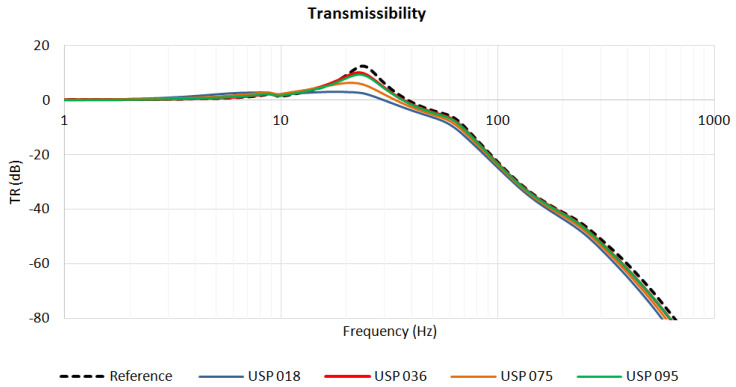
Transmissibility: reference system and systems with four analyzed USP.

**Figure 6:**
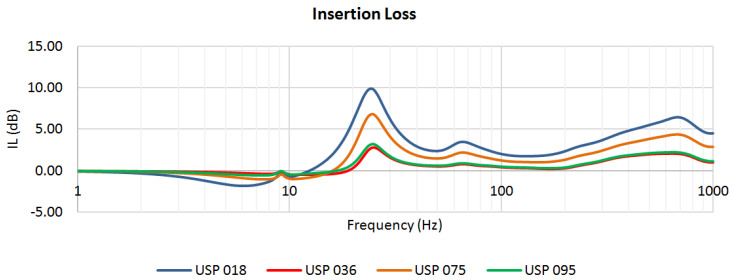
Insertion Loss: systems with four analyzed USP.

The described numerical simulations enabled the authors to find the static and dynamic characteristics of the analyzed structures with resilient USP. For each of the four considered pad materials, three characteristics were determined:

- magnitude of vertical displacement of the left rail head in the frequency domain (Figure 4),
- transmissibility function (Figure 5),
- insertion loss function (Figure 6).

Figure 4 and Figure 5 contain, apart from the curves obtained for systems with USP, the characteristics determined for the reference system, which is a system without USP. The transmissibility function for the reference model was determined as:

(4) $$H_{\mathrm{vib}}\left(i\omega \right)=\frac{R_{\mathrm{vib}}\left(i\omega \right)}{F_{0}},$$

where $R_{\mathrm{vib}}\left(i\omega \right)$ is the subsoil reaction in the reference model, and $F_{0}=2\;N$ is the initial value of the vertical load applied to the rail heads.

Similarly, the transmissibility functions for the systems with USP were calculated:

(5) $$IL\left(\omega \right)=20\cdot \log_{10}\left|\frac{H_{\mathrm{ref}}^{}\left(i\omega \right)}{H_{\mathrm{vib}}^{}\left(i\omega \right)}\right|\;\mathrm{dB},$$

where $R_{\mathrm{vib}}\left(i\omega \right)$ is the subsoil reaction in the vibro-isolated model.

The insertion loss function was determined using the following formula:

(6) $$IL\left(\omega \right)=20\cdot \log_{10}\left|\frac{H_{\mathrm{ref}}^{}\left(i\omega \right)}{H_{\mathrm{vib}}^{}\left(i\omega \right)}\right|\;\mathrm{dB}.$$

The plots depicted in Figure 4 can be used to determine an increase of the static displacement between the reference system and the vibro-isolated one, which is an important parameter that is taken into account while designing such structures. The following values were obtained: USP 018—5.9 mm, USP 036—0.56 mm, USP 075—2.17 mm and USP 095—1.29 mm. Based on these values, the pad USP 018 can be classified as very soft, USP 075 as soft, USP 095 as medium and USP 036 as stiff—the classification is consistent with the one based on the laboratory results.

The curves depicted in Figure 6 indicate a region of effective vibration isolation where IL>0. For USP 018, it starts from 12.4 Hz, USP 036—18.6 Hz, USP 075—15.9 Hz and USP 095—16.7 Hz. The softer the pad is, the bigger the region of vibration isolation effectiveness is.

## 4. Mechanical Model of the Structure

Typically, in the analyses of the influence of vibration isolators on the behavior of railway track structures loaded with harmonic forces, discrete mechanical models with few degrees of freedom are used. Such systems consist of a finite number of material points, springs, dashpots or other elements reflecting the inertial properties of the structure (material points or solids) and the properties connected with the energy accumulation (springs) and dissipation (dashpots).

In the DIN standard [20], a simple discrete model consisting of one material point with $m_{\mathrm{eff}}$ is adopted, and that point characterizes a resultant inertia of all elements of the structure within the vertical oscillation. In the proposed model, the elastic and dissipative properties are modeled using simple rheological Kelvin–Voigt (KV) systems (spring-dashpot systems in parallel). A two-level KV system is assumed, where the upper level corresponds to the properties of the ballast and rail pad (parameters $k_{0}$ and $d_{0}$) and the lower level characterizes the subsoil properties (parameters $k_{u}$ and $d_{u}$). Such a model is called a reference system. In order to take into account the vibration isolators such as USP, a new level of the rheological system needs to be introduced. In the mentioned standard [20], the vibro-isolated KV system contains an additional level with the parameters $k_{\mathrm{el}}\;\left(N/m\right)$ and $d_{\mathrm{el}}\;\left(\mathrm{Ns}/m\right)$.

**Figure 7:**
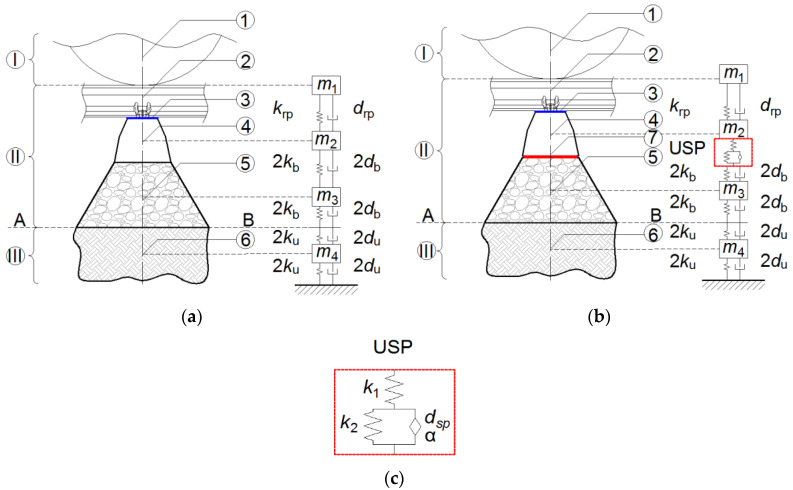
Rheological model of the ballasted track structure: (**a**) Reference model; (**b**) Model with USP; (**c**) Rheological model of USP. Symbols: A-B—subgrade surface layer; I—rolling stock, II—railway track structure, III—subgrade; 1—non-spring part of the rolling stock, 2—rail, 3—rail pad, 4—sleeper, 5—ballast, 6—subgrade, 7—USP.

In the present paper, the authors present a novel approach to the analysis of ballasted track structures with resilient under sleeper pads. The proposed mechanical model describes precisely the structural behavior and is more accurate than the standard system presented in [20]. It has four degrees of freedom (4DoF) corresponding to four material points with the following masses: $m_{1}$—rail and rolling stock, $m_{2}$—sleeper, $m_{3}$—ballast and $m_{4}$—soil subgrade (see Figure 7). In the developed 4DoF model, the elastic and dissipative elements, modeled with rheological KV systems, characterize the properties of: the rail pad ($k_{\mathrm{rp}}$ and $d_{\mathrm{rp}}$), ballast ($k_{b}$ and $d_{b}$) and subsoil ($k_{u}$ and $d_{u}$). The reference model is presented in Figure 7a. The isolated system contains an additional level representing the under sleeper pad (Figure 7b). The rheological viscoelastic model of USP is shown in Figure 7c. It is a fractional Zener model (FZM), which is composed of two springs, $k_{1}$ and $d_{2}$, and a fractional element (spring-pot) defined by two parameters, $d_{\mathrm{sp}}$ and α.

The proposed FZM model has not been considered in the literature in relation to USP before. Using this model, the curves of the dynamic bedding moduli can be determined, which are consistent with the ones obtained from the laboratory tests. It gives much more accurate results than other tested rheological models (e.g., the Burger model, Prony series and others).

The dynamic characteristic of the 4DoF system, for example in the form of a complex stiffness function, can be formulated analytically using the Fourier transform. It should be noted that in the presented formulation the 4DoF model needs to be treated as a SIMO (Single Input Multiple Output) system, where the input is a harmonic function $F\left(t\right)=f_{o}\;e^{i\omega \;t}$ with an amplitude $f_{o}=1\;N$ and a random frequency ω [rad/s] applied to the rail head. The output values are the displacements and velocities corresponding to the four degrees of freedom of the system. From such a defined mechanical model, four complex stiffness functions $K_{ij}^{*}$ are obtained, where i is an input number (in this case i=1), and j corresponds to an output value. The following output numbering is used: 1—rail head, 2—sleeper, 3—ballast and 4—subsoil.

It should be highlighted that the proposed methodology is contained within the theory of linear dynamic systems. The obtained results characterize the response of the structure in the form of an amplitude and a phase angle in the steady-state harmonic oscillation. The following expressions describing the complex stiffness functions can be formulated for the reference model:

(7) $$K_{11}^{*}=k_{1}^{*},$$

(8) $$K_{12}^{*}=k_{2}^{*}{\left(1+\frac{m_{1}\;\omega^{2}}{K_{11}^{*}}\right)}^{-1},$$

(9) $$K_{13}^{*}=k_{3}^{*}{\left(1+\frac{m_{1}\;\omega^{2}}{K_{11}^{*}}+\frac{m_{2}\;\omega^{2}}{K_{12}^{*}}\right)}^{-1},$$

(10) $$K_{14}^{*}=k_{4}^{*}{\left(1+\frac{m_{1}\;\omega^{2}}{K_{11}^{*}}+\frac{m_{2}\;\omega^{2}}{K_{12}^{*}}+\frac{m_{3}\;\omega^{2}}{K_{13}^{*}}\right)}^{-1},$$

where:

(11) $$k_{4}^{*}:=-m_{4}\;\omega^{2}+2k_{u}+2d_{u}\;i\;\omega ,$$

(12) $$k_{3}^{*}:=-m_{3}\;\omega^{2}+{\left(\frac{1}{k_{\mathrm{bu}}+d_{\mathrm{bu}}\;i\;\omega }+\frac{1}{k_{4}^{*}}\right)}^{-1},$$

(13) $$k_{2}^{*}:=-m_{2}\;\omega^{2}+{\left(\frac{1}{2k_{b}+2d_{b}\;i\;\omega }+\frac{1}{k_{3}^{*}}\right)}^{-1},$$

(14) $$k_{1}^{*}:=-m_{1}\;\omega^{2}+{\left(\frac{1}{k_{\mathrm{rp}}+d_{\mathrm{rp}}\;i\;\omega }+\frac{1}{k_{2}^{*}}\right)}^{-1},$$

(15) $$k_{\mathrm{bu}}:=\frac{2k_{b}\;k_{u}}{k_{b}+k_{u}},\;d_{\mathrm{bu}}:=\frac{2d_{b}\;d_{u}}{d_{b}+d_{u}}.$$

The parameters applied in the reference 4DoF model are given in Table 3.

The presented relationships can be further developed by adding the terms that correspond to the complex stiffness functions of the vibration isolators (USP). The constitutive relationships of the fractional element (spring-pot), relating the force f(t) and the displacement $u_{\mathrm{sp}}\left(t\right)$, may be expressed in differential form:

(16) $$f\left(t\right):=d_{\mathrm{sp}}\;D^{\alpha }u_{\mathrm{sp}}\left(t\right)\;;\;\alpha \in \left(0,\;1\right),$$

where $D^{\alpha }\equiv \frac{d^{\alpha }}{d\;t^{\alpha }}$ denotes the fractional derivative operator:

(17) $$\begin{array}{c} D^{\alpha }u_{sp}\left(t\right):=\frac{u_{sp}\left(0\right)}{\Gamma \left(1-\alpha \right)\;\;t^{\alpha }}\\ +\frac{1}{\Gamma \left(1-\alpha \right)}\;\;\int_{0}^{t}\frac{{\dot{u}}_{sp}\left(\tau \right)}{{\left(\;t-\tau \right)}^{\alpha }}\;d\tau \end{array}$$

(18) $$\Gamma \left(1-\alpha \right):=\int_{0}^{\infty }t^{-\alpha }e^{-t}d\;t.$$

**Figure 8:**
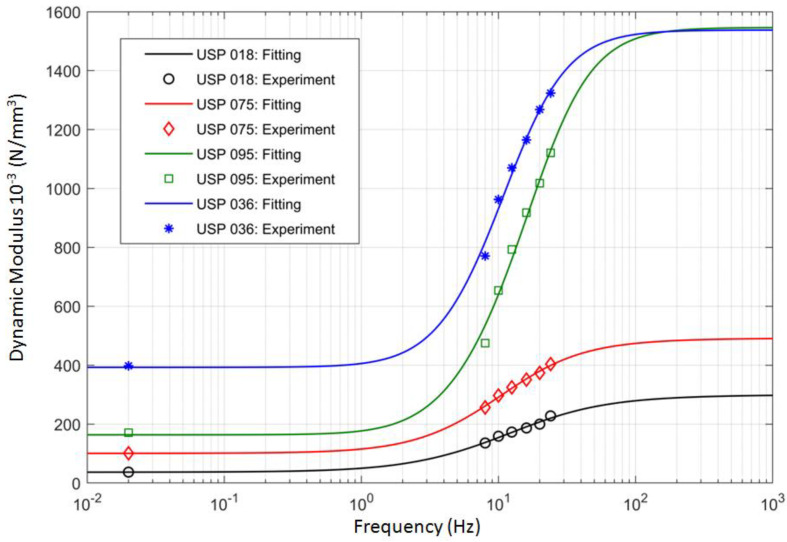
Curve fitting results for the four analyzed USP models.

When α=1, a classical dashpot model is obtained; taking α=0 leads to a simple spring element. Based on the results of the laboratory tests presented in the previous sections, the rheological parameters of the four analyzed under sleeper pads can be determined using a curve-fitting procedure. The results of this procedure, implemented in Matlab software (Matlab 2018b), are visualized in Figure 8. The curve fitting algorithm is based on the calculation of the dynamic stiffness moduli, which are the absolute values of the complex stiffness $k_{\mathrm{USP}}^{*}$.

One can note that the curves (Figure 8) become stable after a certain frequency. This is a typical behavior of materials with viscoelastic properties [22]. Dynamic stiffness moduli are often represented using logistic functions. Such an approach is used, for example, in the case of mineral–asphalt mixtures. In the present paper, another approach is applied—the system is analyzed using rheological models, which make it possible to define analytical formulae for the complex stiffness and, afterwards, to determine numerically its magnitude and phase. The analytical formulation of the complex stiffness allowed the authors to include the USP model in the 4DoF model, which would not be possible with the use of the logistic function.

The complex stiffness of the USP model $k_{\mathrm{USP}}^{*}$ and of the entire isolated system $k_{\mathrm{vib}}^{*}$ can be defined as follows:

(19) $$k_{\mathrm{USP}}^{*}\left(i\omega \right)={\left[\frac{1}{k_{1}}+\frac{1}{k_{2}+d_{\mathrm{sp}}{\left(i\;\omega \right)}^{\alpha }}\right]}^{-1},$$

(20) $${k_{\mathrm{vib}}^{*}\left(i\omega \right)=\left[\frac{1}{k_{\mathrm{ref}}^{*}\left(i\omega \right)}+\frac{1}{k_{\mathrm{USP}}^{*}\left(i\omega \right)}\right]}^{-1}.$$

It is also possible to determine complex transfer functions for the reference system $H_{ref}^{*}\left(i\omega \right)$ and the isolated one $H_{vib}^{*}\left(i\omega \right)$:

(21) $$H_{\mathrm{ref}}^{*}\left(i\omega \right)=\frac{k_{\mathrm{ref}}^{*}\left(i\omega \right)}{k_{\mathrm{ref}}^{*}\left(i\omega \right)-m_{\mathrm{eff}}\;\omega^{2}},$$

(22) $$H_{\mathrm{vib}}^{*}\left(i\omega \right)=\frac{k_{\mathrm{vib}}^{*}\left(i\omega \right)}{k_{\mathrm{vib}}^{*}\left(i\omega \right)-m_{\mathrm{eff}}\;\omega^{2}}.$$

The absolute values of the complex transfer functions can be interpreted as the transmissibility of a harmonically excited system.

Moreover, using the above definitions, an insertion loss parameter can be evaluated:

(23) $$IL\left(\omega \right)=20\cdot \log_{10}\left|\frac{H_{\mathrm{ref}}^{*}\left(i\omega \right)}{H_{\mathrm{vib}}^{*}\left(i\omega \right)}\right|\;\mathrm{dB}.$$

**Figure 9:**
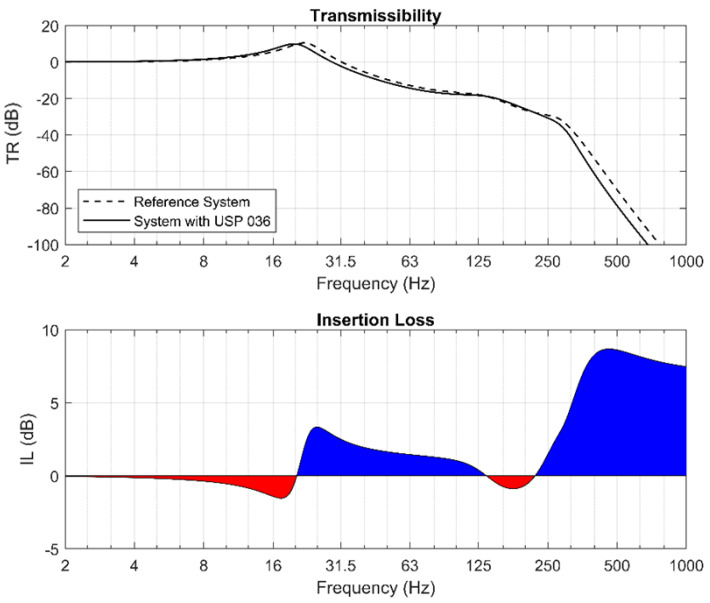
Transmissibility and insertion loss: reference system and system with USP 036.

The calculations performed with the use of a program developed within the Matlab system, which is based on the methodology presented above, allowed the authors to obtain the dynamic characteristics of the considered structures with vibration isolators. In Figure 9, the transmissibility and insertion loss functions determined for USP 036 are presented. The insertion loss diagram indicates a low effectiveness of vibration isolation for frequencies below 125 Hz. Moreover, the application of the analyzed pad can even deteriorate the vibration isolation capacity in the systems excited at frequencies of ca. 125–250 Hz (negative values of insertion loss are marked in red).

One can note that the results obtained from the mechanical model of the structure are consistent with the ones from the FE analysis. This proves that the proposed 4DoF rheological model of vibro-isolated ballasted track structures retrofitted with USP is highly precise and can be used to determine the dynamic characteristics of the systems and to indicate the regions of effective vibration isolation.

## 5. Conclusions

In the present paper, an original approach to the analysis of vibro-isolated ballasted track systems was proposed. In the first part, the authors presented the results of laboratory tests which were carried out to determine the static and dynamic bedding moduli of the analyzed under sleeper pads (USP). The values obtained from these tests were afterwards used as an input for the finite element (FE) and mechanical models of the structure. For the purpose of this paper, four different USP samples were considered: two rubber-based and two polyurethane-based pads differing in thickness.

The FE model allowed the authors to find the static and dynamic characteristics of the analyzed structures with resilient USP. For each of the four considered pad materials, three characteristics were determined: the magnitude of vertical displacement of the left rail head in the frequency domain, transmissibility function and insertion loss function. Based on the results of the FE analysis, the following information was obtained: the increment of static displacement between the reference system and the vibro-isolated one, which is an important parameter that is taken into account while designing such structures; the classification of the pads as soft, medium or stiff; the effectiveness of the vibration isolation. The polyurethane-based pad USP 018 turned out to be the softest out of the four considered samples—the static displacement increased by 5.9 mm, and it exhibited the widest region of effective vibration isolation (from 12.4 Hz). The styrene–butadiene rubber-based pad USP 036, on the other hand, was the stiffest one, with an increment of static displacement around ten times smaller than for USP 018 (0.56 mm) and with the smallest region of isolation effectiveness (starting from 18.6 Hz).

A special focus was put on the description of an original four-degree-of-freedom (4DoF) mechanical model of the system that includes a fractional rheological model of USP. Typically, in the analyses of railway track structures with vibration isolators, discrete mechanical models with few degrees of freedom are applied. In the present paper, the authors proposed a mechanical model that has four degrees of freedom corresponding to four material points: rail and rolling stock, sleeper, ballast and soil subgrade. The elastic and dissipative elements are modeled with rheological Kelvin–Voigt systems, which characterize the properties of the rail pad, ballast and subsoil. Moreover, the isolated system contains an additional level representing the under sleeper pad, whose rheological viscoelastic model is composed of two springs and a fractional element (spring-pot). The proposed mechanical model of the ballasted track system describes precisely the behavior of the structure and is more accurate than standard systems described in the literature.

## Figures and Tables

**Table 1:** The static and dynamic bedding moduli of the four tested under sleeper pads (USP).

Parameter		USP 018	USP 036	USP 075	USP 095
$C_{\mathrm{stat}}\;\left[N/{\mathrm{mm}}^{3}\right]$		0.037	0.398	0.101	0.171
$C_{\mathrm{dyn}}\;\left[N/{\mathrm{mm}}^{3}\right]$	8 Hz	0.136	0.771	0.257	0.475
	10 Hz	0.159	0.963	0.297	0.654
	12.5 Hz	0.173	1.070	0.325	0.793
	16 Hz	0.187	1.165	0.351	0.918
	20 Hz	0.200	1.268	0.374	1.018
	24 Hz	0.228	1.324	0.404	1.121

**Table 2:** Material parameters used in the FE model.

Layer	E [GPa]	$\rho \;\left[\mathrm{kg}/m^{3}\right]$	ν	η
Rail	200	7850	0.3	0.35
Sleeper	3.2	1250	0.2	0.35
Ballast	0.3	2000	0.2	0.35
Protection layer	0.15	2000	0.2	0.61
Soil subgrade	0.035	2000	0.3	0.61

**Table 3:** Parameters of the 4DoF model—reference system.

No.	Layer	m(kg)	k (N/m)	d (Ns/m)	η
1	Rail + rail pad + rolling stock	$m_{1}=3351$	$k_{\mathrm{rp}}=1.00\times 10^{8}$	$d_{\mathrm{rp}}=1.0\times 10^{5}$	$\eta_{\mathrm{rp}}=0.05$
2	Sleeper	$m_{2}=165$	-	-	-
3	Ballast	$m_{3}=422$	$k_{b}=2.50\times 10^{8}$	$d_{b}=0$	$\eta_{b}=0.35$
4	Soil subgrade	$m_{4}=603$	$k_{u}=2.50\times 10^{8}$	$d_{u}=0$	$\eta_{u}=0.30$

## Nomenclature

$C_{\mathrm{stat}}\;$	static bedding modulus
$C_{\mathrm{dyn}}$	dynamic bedding modulus
E	elastic modulus
ρ	density
ν	Poisson’s ratio
η	loss factor
k	stiffness coefficient
$d_{}$	damping coefficient
m	mass
$m_{\mathrm{eff}}$	effective mass
$A_{\mathrm{eff}}$	effective area
f	frequency
$F_{0}$	force
H(iω)	transmissibility function
R(iω)	subsoil reaction function
IL(ω)	insertion loss function
$K_{ij}^{*}$	complex stiffness functions
f(t)	force function
u(t)	displacement function
$H_{}^{*}\left(i\omega \right)$	complex transfer function
$D^{\alpha }\equiv \frac{d^{\alpha }}{d\;t^{\alpha }}$	fractional derivative operator

## References

[1] Marschnig S., Veit P. (2011). Making a case for under sleeper pads. Int. Railw. J..

[2] Kraśkiewicz C., Zbiciak A., Al Sabouni-Zawadzka A., Piotrowski A. (2020). Experimental research on fatigue strength of prototype under sleeper pads used in the ballasted rail track systems. Arch. Civ. Eng..

[3] Sol-Sánchez M., Moreno-Navarro F., Rubio-Gámez C. (2015). The use of elastic elements in railway tracks: A state of the art review. Constr. Build. Mater..

[4] Sol-Sánchez M., Pirozzolo L., Moreno-Navarro F., Rubio-Gámez C. (2016). A study into the mechanical performance of different configurations for the railway track section: A laboratory approach. Eng. Struct..

[5] Sol-Sánchez M., Moreno-Navarro F., Rubio-Gámez C. (2014). The use of deconstructed tires as elastic elements in railway tracks. Materials.

[6] Sol-Sánchez M., Thom N.H., Moreno-Navarro F., Rubio-Gámez C., Airey G.D. (2015). A study into the use of crumb rubber in railway balast. Constr. Build. Mater..

[7] Kennedy J., Woodward P.K., Medero G., Banimahd M. (2013). Reducing railway track settlement using three-dimensional polyurethane polymer reinforcement of the ballast. Constr. Build. Mater..

[8] Kaewunruen S., Aikawa A., Remennikov A.M. (2017). Vibration attenuation at rail joints through under sleeper pads. Procedia Eng..

[9] Omodaka A., Kumakura T., Konishi T. (2017). Maintenance reduction by the development of resilient sleepers for ballasted track with optimal under-sleeper pads. Procedia CIRP.

[10] Abadi T., Le Pen L., Zervos A., Powrie W. (2019). Effect of sleeper interventions on railway track performance. J. Geotech. Geoenviron. Eng..

[11] Jayasuriya C., Indraratna B., Ngo T.N. (2019). Experimental study to examine the role of under sleeper pads for improved performance of ballast under cyclic loading. Transp. Geotech..

[12] Zbiciak A., Kraśkiewicz C., Oleksiewicz W., Lipko C. Viscoelastic dynamic models of resilient elements used in railway tracks. Proceedings of the MATEC Web of Conferences, 5th International Scientific Conference “Integration, Partnership and Innovation in Construction Science and Education”.

[13] Zbiciak A., Kraśkiewicz C., Oleksiewicz W., Płudowska-Zagrajek M., Lipko C. Mechanical modelling and application of vibroacoustic isolators in railway tracks. Proceedings of the MATEC Web of Conferences, RSP 2017–XXVI R-S-P Seminar 2017 Theoretical Foundation of Civil Engineering.

[14] Grzesikiewicz W., Wakulicz A., Zbiciak A. (2013). Non-linear problems of fractional calculus in modeling of mechanical systems. Int. J. Mech. Sci..

[15] Grzesikiewicz W., Osiecki J., Piotrowski J. (1974). Podstawy dynamiki pojazdów szynowych.

[16] Wettschureck R.G. (1987). Unterschottermatten auf einer Eisenbahnbrücke in Stahlbeton-Verbundbauweise. DAGA.

[17] Wettschureck R.G., Heim M., Mühlbachler S. (1997). Reduction of structure-borne noise emissions from above-ground railway lines by means of ballast mats. INTER-NOISE NOISE-CON Congr. Conf. Proc..

[18] Sołkowski J. (2015). Efektywność wibroizolacji nawierzchni kolejowej w ujęciu analitycznym. Zeszyty Naukowo-Techniczne Stowarzyszenia Inżynierów i Techników Komunikacji w Krakowie. Seria: Materiały Konferencyjne.

[19] (2016). PN-EN 16730:2016-08 Railway Applications—Track—Concrete Sleepers and Bearers with Under Sleeper Pads.

[20] (2008). DIN 45673-4:2008-07 Mechanical Vibration. Resilient Elements Used in Railway Tracks. Part 4: Analytical Evaluation of Insertion Loss of Mounted Track Systems.

[21] (2018). DIN 45672-1:2018-02 Vibration Measurement Associated with Railway Traffic Systems. Part 1: Measuring Method for Vibration.

[22] Christensen R.M. (1982). Theory of Viscoelasticity. An Introduction.

